# Predicting Hypertension Subtypes with Machine Learning Using Targeted Metabolites and Their Ratios

**DOI:** 10.3390/metabo12080755

**Published:** 2022-08-16

**Authors:** Smarti Reel, Parminder S. Reel, Zoran Erlic, Laurence Amar, Alessio Pecori, Casper K. Larsen, Martina Tetti, Christina Pamporaki, Cornelia Prehn, Jerzy Adamski, Aleksander Prejbisz, Filippo Ceccato, Carla Scaroni, Matthias Kroiss, Michael C. Dennedy, Jaap Deinum, Graeme Eisenhofer, Katharina Langton, Paolo Mulatero, Martin Reincke, Gian Paolo Rossi, Livia Lenzini, Eleanor Davies, Anne-Paule Gimenez-Roqueplo, Guillaume Assié, Anne Blanchard, Maria-Christina Zennaro, Felix Beuschlein, Emily R. Jefferson

**Affiliations:** 1 Division of Population Health and Genomics, School of Medicine, University of Dundee, Dundee DD2 4BF, UK; 2 Diabetologie und Klinische Ernährung, Klinik für Endokrinologie, UniversitätsSpital Zürich (USZ) und Universität Zürich (UZH), CH-8091 Zurich, Switzerland; 3 Université Paris Cité, INSERM, PARCC, F-75015 Paris, France; 4 Unité Hypertension Artérielle, Assistance Publique-Hôpitaux de Paris, Hôpital Européen Georges Pompidou, F-75015 Paris, France; 5 Division of Internal Medicine and Hypertension Unit, Department of Medical Sciences, University of Torino, 10124 Torino, Italy; 6 Department of Medicine III, Universitätsklinikum Carl Gustav Carus, Technische Universität, 01307 Dresden, Germany; 7 Metabolomics and Proteomics Core (MPC), Helmholtz Zentrum München, German Research Center for Environmental Health, 85764 Neuherberg, Germany; 8 Institute of Experimental Genetics, Helmholtz Zentrum München, German Research Center for Environmental Health, Ingolstädter Landstraße 1, 85764 Neuherberg, Germany; 9 Department of Biochemistry, Yong Loo Lin School of Medicine, National University of Singapore, 8 Medical Drive, Singapore 117597, Singapore; 10 Institute of Biochemistry, Faculty of Medicine, University of Ljubljana, Vrazov trg 2, 1000 Ljubljana, Slovenia; 11 Department of Hypertension, National Institute of Cardiology, 04-628 Warsaw, Poland; 12 UOC Endocrinologia, Dipartimento di Medicina DIMED, Azienda Ospedaliera-Università di Padova, 35128 Padua, Italy; 13 Clinical Chemistry and Laboratory Medicine, Core Unit Clinical Mass Spectrometry, Universitätsklinikum Würzburg, 97080 Würzburg, Germany; 14 Schwerpunkt Endokrinologie/Diabetologie, Medizinische Klinik und Poliklinik I, Universitätsklinikum Würzburg, 97080 Würzburg, Germany; 15 Comprehensive Cancer Center Mainfranken, Universität Würzburg, 97070 Würzburg, Germany; 16 Medizinische Klinik und Poliklinik IV, Klinikum der Universität München, LMU München, 80336 Munich, Germany; 17 The Discipline of Pharmacology and Therapeutics, School of Medicine, National University of Ireland 33 Galway, H91 TK33 Galway, Ireland; 18 Department of Medicine, Section of Vascular Medicine, Radboud University Medical Center, 6525 GA Nijmegen, The Netherlands; 19 Department of Medicine III and Institute of Clinical Chemistry and Laboratory Medicine, Universitätsklinikum Carl Gustav Carus, 01307 Dresden, Germany; 20 Internal & Emergency Medicine, ESH Specialized Hypertension Center, Department of Medicine-DIMED, University of Padua, 35128 Padua, Italy; 21 Institute of Cardiovascular & Medical Sciences, BHF Glasgow Cardiovascular Research Centre, University of Glasgow, Glasgow G12 8TA, UK; 22 Service de Génétique, Assistance Publique-Hôpitaux de Paris, Hôpital Européen Georges Pompidou, F-75015 Paris, France; 23 Institut Cochin, Université de Paris, INSERM, CNRS, F-75014 Paris, France; 24 Department of Endocrinology, Center for Rare Adrenal Diseases, Assistance Publique–Hôpitaux de Paris, Hôpital Cochin, F-75014 Paris, France; 25 Centre d’Investigations Cliniques 9201, Assistance Publique-Hôpitaux de Paris, Hôpital Européen Georges Pompidou, F-75015 Paris, France; 26 Institute of Health & Wellbeing, University of Glasgow, Glasgow G12 8RZ, UK

**Keywords:** metabolomics, machine learning, hypertension, primary aldosteronism, pheochromocytoma/paraganglioma, Cushing syndrome, biomarkers

## Abstract

Hypertension is a major global health problem with high prevalence and complex associated health risks. Primary hypertension (PHT) is most common and the reasons behind primary hypertension are largely unknown. Endocrine hypertension (EHT) is another complex form of hypertension with an estimated prevalence varying from 3 to 20% depending on the population studied. It occurs due to underlying conditions associated with hormonal excess mainly related to adrenal tumours and sub-categorised: primary aldosteronism (PA), Cushing’s syndrome (CS), pheochromocytoma or functional paraganglioma (PPGL). Endocrine hypertension is often misdiagnosed as primary hypertension, causing delays in treatment for the underlying condition, reduced quality of life, and costly antihypertensive treatment that is often ineffective. This study systematically used targeted metabolomics and high-throughput machine learning methods to predict the key biomarkers in classifying and distinguishing the various subtypes of endocrine and primary hypertension. The trained models successfully classified CS from PHT and EHT from PHT with 92% specificity on the test set. The most prominent targeted metabolites and metabolite ratios for hypertension identification for different disease comparisons were C18:1, C18:2, and Orn/Arg. Sex was identified as an important feature in CS vs. PHT classification.

## 1. Introduction

One of the main risk factors for cardiovascular disease is arterial hypertension. Arterial hypertension is a significant health problem that affects a wide population every year [1]. The underlying mechanisms of primary (essential) arterial hypertension are multiple and largely unknown. There are forms of so-called secondary hypertension, where arterial hypertension is one of the clinical manifestations of the underlying disease. Among those, we distinguish the endocrine hypertension cases, caused by hormonal hypersecretion mainly related to diseases of the adrenal glands. The latter are represented by primary aldosteronism (PA), Cushing’s syndrome (CS), and pheochromocytoma/functional paraganglioma (PPGL), which are highly challenging to diagnose in the early stages [2]. The reason for this lies in the cumbersome diagnostic process, requiring complex pre-analytical procedures and expertise in the interpretation of the test results, making it less available for the high number of patients of this global pandemic. Metabolomics has already been successfully used in patients with endocrine-related hypertension [3,4,5] and recently our research group identified different metabolic fingerprint discrimination between primary and endocrine hypertension cases [6]. Metabolomics is a relatively new approach for the parallel and high-throughput identification and quantification of numerous low molecular weight molecules (metabolites). Whilst untargeted metabolomics identifies numerous molecules without prior knowledge of their presence, there is often a lack of quantification and definite biochemical annotation. In contrast, targeted metabolomics provides the advantage of reliable quantification of metabolites with known biochemical annotation making it more suitable for the diagnostic purpose [7].

Machine learning (ML) is capable of processing large datasets in a minimal time frame and can provide accurate clinical insights to aid physicians in diagnosis and treatments. In recent years, ML methods have been widely popular in medicine [8,9], biomarker discovery in high-dimensional omics data [10], and detecting signatures of disease in liquid biopsies [11]. Some studies investigated targeted metabolomics markers of preclinical Alzheimer’s disease [12], psoriasis [13], and the detection of intrauterine growth restriction [14]. In the past, a variety of ML methods such as *k*-nearest neighbours, support vector machines, and decision trees have been evaluated for targeted metabolomics [15,16].

In this study, we investigated various supervised machine learning methods and evaluate their classification performance through overall classification accuracy, specificity, and sensitivity using the targeted metabolomics dataset previously published [6]. The dataset was also investigated within subsets of age and sex to evaluate its impact on the model training, prediction performance, and corresponding selected features. The most prominent metabolites and their ratios were identified for distinguishing various hypertension subtypes.

## 2. Materials and Methods

### 2.1. Omic Dataset

The metabolomics dataset was described in detail in our previous work [6]. Briefly, blood plasma samples were collected from 294 male and female patients between 16–78 years with one of the four underlying hypertension subtypes, (PA, PPGL, CS, and PHT). Of the 282 patients included in the final analyses (see the exclusion of outliers below), we had information on the presence of diabetes mellitus in 88.7% and BMI data for 86.9% of cases. Diabetes mellitus was present in 12% of cases, with a higher prevalence in patients with CS (26.7%) and PPGL (26.5%), as expected [17,18,19]. Obesity (BMI ≥ 30 kg/m^2^) was present in 24.5% of patients, with the highest prevalence in patients with CS (40%), followed by PA (32.6%), PHT (22.4%), and PPGL (7.7%), in accordance with the literature [17,18,19]. The PA patients comprised of aldosterone-producing adenoma (APA) (*n* = 66), bilateral adrenal hyperplasia (BAH) (*n* = 36), and unknown (*n* = 5, adrenal venous sampling failed: 1 and refused: 4). The samples were provided by 11 centers of the ENS@T-HT consortium (http://www.ensat-ht.eu accessed on 1 June 2022). The study was conducted according to the guidelines of the Declaration of Helsinki and approved by the local ethics committees of participating centers.

Table 1 presents a breakdown of the patients by their disease subtypes for analysis, after the exclusion of outliers (see below). The specific inclusion and exclusion criteria for each hypertension subtype are provided in Appendix B.

The targeted metabolomics approach was based on LC-ESI-MS/MS and FIA-ESI-MS/MS measurements by Absolute*IDQ*^TM^ p180 Kit (BIOCRATES Life Sciences AG, Innsbruck, Austria). The assay allows simultaneous quantification of 188 metabolites and includes free carnitine, 39 acylcarnitines, 21 amino acids (19 proteinogenic + citrulline + ornithine), 21 biogenic amines, hexoses (sum of hexoses—about 90–95% glucose), 90 glycerophospholipids (14 lysophosphatidylcholines (lysoPC) and 76 phosphatidylcholines (PC)), and 15 sphingolipids (SM). Further details are provided in Appendix C.

In addition to the investigated samples, five aliquots of a pooled reference plasma were analysed on each kit plate. The results of these reference plasma aliquots were used for the calculation of potential batch effects and data normalization. We included all metabolite measurements with peaks above the limit of detection, defined as three times the values of the zero samples, as well as those below this threshold if the respective peak was detectable visually. To ensure the comparability of received data between batches, each metabolite value was normalized as previously described [20,21]. Metabolites for which measurement values were valid in less than 3 of 5 reference plasma were excluded from normalization and further statistical analysis. We further excluded metabolites for which the coefficient of variance of reference plasma was >25% within and between batches (exceptions included 8 metabolites for which only the variance between batches, but not within, were only slightly above the predetermined cut-off prior normalization) and those metabolites for which values were not detectable in >40% of samples. From 188 metabolites, 155 passed these selection criteria. In addition to the 155 eligible metabolites, 18 pre-defined metabolite sums and ratios were eligible for further analyses (See Table A1 in Appendix A). The missing values of the metabolites with <40% of undetectable data were estimated using the KNN method, considering each subgroup of clinical conditions separately [22].

Using the heatmap analysis method, we identified potential outliers among the studied patients as previously described [23], and those patients were excluded from the statistical analysis. In total, 282 patients were eligible for further analyses (See Table 1).

The missing data estimation and outlier detection were performed using the MetaboAnalyst platform [23]. The final dataset was catalogued in RDMP Software [24] for systematic access.

### 2.2. ML Analysis Pipeline

**Figure 1:**
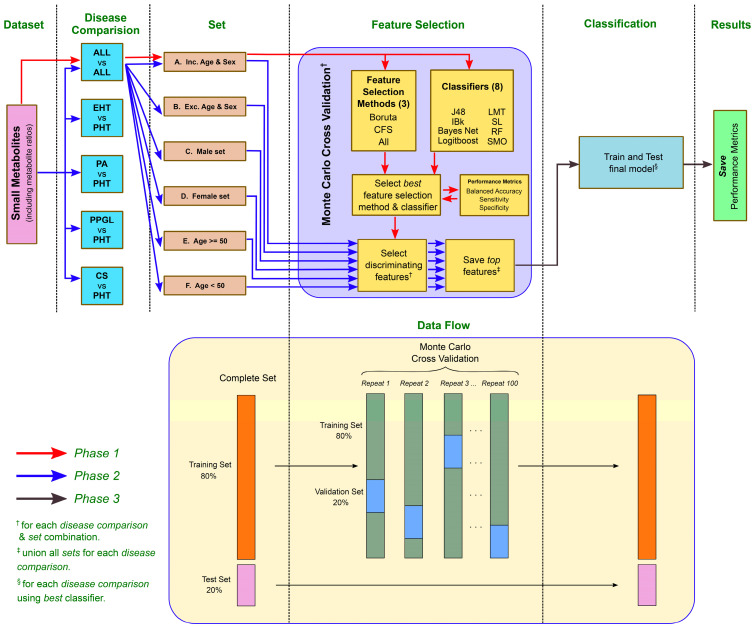
ML analysis pipeline showing the three phases of the analysis and corresponding data flow.

The small metabolites data was evaluated for five different *disease comparisons* namely All vs. All (i.e., PA vs. PPGL vs. CS vs. PHT), EHT (i.e., PA + PPGL + CS) vs. PHT, PA vs. PHT, PPGL vs. PHT, and CS vs. PHT (See Figure 1). Each of these comparisons was investigated for possible bias due to age and sex by creating six sets. These sets included: A. All patients, all metabolite features (including age and sex); B. All patients, all metabolite features (excluding age and sex); C. Male patients, all metabolite features (including age); D. Female patients, all metabolite features (including age); E. All patients (with age ≥ 50 years), all metabolite features (including sex); and F. All patients (with age < 50 years), all metabolite features (including sex). Set E and F were bifurcated based on average female menopausal age i.e., 50 years to understand the effect of patient age on metabolites. These segregated sets were also useful in comparing their respective significant discriminating features and using them for final model training.

The ML analysis pipeline investigated (See Figure 1) three feature selection methods: (a) Using all features, (b) CFS: correlation-based feature selection [25], and (c) Boruta [26]; and eight different supervised learning classifiers (J48 [27], IBk [28], Bayes Net [29], Logitboost [30], Logistic Model Tree (LMT) [31], Simple Logistic (SL) [32], Random Forest (RF) [33], and Sequential minimal optimization (SMO) [34]).

The complete metabolomics dataset was randomly partitioned into 80% training and 20% testing sets (See Table A2 in Appendix A). The training set was used for the Monte Carlo Cross-Validation (MCCV) approach [35] and, therefore, further partitioned into 80% training and 20% validation sets. On the other hand, the testing set was only used to test the final model (See Figure 1). A set of five metrics: balanced accuracy (arithmetic mean between sensitivity and specificity) [36], sensitivity, specificity, F1 score (with beta = 1), and AUC were used to evaluate the classification performance. These were calculated using the confusionMatrix function from caret package [37].

The ML analysis pipeline was divided into three phases. Phase 1 studied the best feature selection and top classification algorithms using All vs. All disease comparison for set *A* (as they represent the complete dataset) with the MCCV approach. It used 100 random repeats (as in [38]) to train algorithms and then compared their average performance metrics (accuracy, sensitivity, and specificity) on the validation set.

In Phase 2, the best feature selection and top 4 classifiers from Phase 1 are used to find the discriminating features (metabolites and their ratios) for remaining disease combinations with MCCV. The most selected features during the 100 random repeats are considered as top features and hence saved.

Finally, in Phase 3, the subset of top common features from the training set was downsampled (to avoid class imbalance) and then used for training the best-performing classifier (from Phase 2). This final classifier was then tested on the test set and the predictions were saved (for each disease comparison and set combination). All classifications were implemented with the RWeka package [39] in the R language [40].

## 3. Results

### 3.1. Evaluation of Feature Selection Methods & Classifiers

Phase 1 of the ML analysis pipeline investigated ALL vs. ALL (PA vs. PPGL vs. CS vs. PHT) disease comparison using CFS and Boruta feature selection methods. The classification was also performed using all features (i.e., no feature reduction). Table 2 shows the mean values of five performance metrics (i.e., balanced accuracy, sensitivity, specificity, F1 score, and AUC) for all three feature selection approaches when used in conjunction with different classifiers across the 100 MCCV repeats. It was observed that using all features for classification provided the best metrics followed by Boruta and CFS methods. Although the mean accuracies for ALL vs. ALL disease comparisons are low, since it is a complex multi-class problem, still it is evident that Boruta being a wrapper-based method provides reasonably better classification than CFS. Table A3, Table A4, Table A5 and Table A6 show the classification performance for the remaining four disease combinations. Hence, Boruta was empirically selected for the rest of the ML analysis pipeline. Similarly, based on the metrics, SL, LMT, LB, and RF were selected as the top four classifiers. RF was selected instead of NB since it was able to provide a consistent performance irrespective of the choice of the feature selection method). Hence, Boruta and SL, LMT, LB, and RF were selected for Phase 2 of the analysis.

### 3.2. Classification Performance and Discriminating Features

In Phase 2 of the analysis, the classification performance and corresponding top discriminating features for the various disease comparisons were individually evaluated.

#### 3.2.1. MCCV Classification Performance

**Figure 2:**
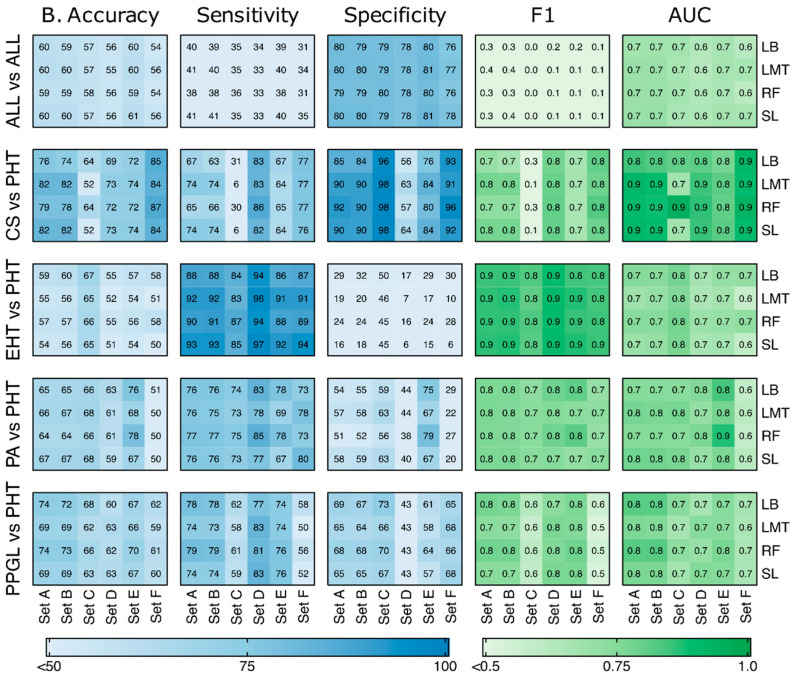
Heatmap comparing accuracy, sensitivity, and specificity for Sets A–F using 5 classifiers for 5 disease combinations (Phase 2). The count in each box is a weighted average of 100 runs (MCCV repeats).

Figure 2 shows mean balanced accuracy, sensitivity, specificity, F1 score, and AUC for five disease comparisons in six sets (A–F) using the top four classifiers with 100 MCCV repeats. The sets were compared as Set A vs. Set B, Set C vs. Set D, and Set E vs. Set F for all five disease comparisons. The non-uniform number of samples in different sets, (e.g., Sets C & D in CS & Set E & F in PPGL) does not validate a direct metric comparison among them, however, it was useful in evaluating the prominent discriminating features in a given disease comparison based on sex and age.

In Set A and Set B, the highest accuracy (~82%) was observed for CS vs. PHT with SL and LMT. The corresponding F1 score and AUC were 0.8 and 0.9 respectively. On the other hand, RF provided the highest specificity (~92%) in CS vs. PHT (Set A). Although EHT vs. PHT had a low accuracy (~54%) and specificity (16%), it still was able to achieve high sensitivity (~93%) using SL in both Set A and B. The corresponding F1 score and AUC were 0.9 and 0.7 respectively. For ALL vs. ALL, SL and LMT achieved higher accuracy (~60%) and specificity (~80%) in comparison to LB and RF. Amongst the two sets, Set A provided better performance for all five metrics irrespective of the classifier used. As earlier in CS vs. PHT, both SL and LMT provided better performance for PA vs. PHT in comparison to RF and LB. For PPGL vs. PHT, LB and RF outperformed LMT and SL. Overall, there is no notable difference in any of the metrics values within Set A and Set B. This shows that age and sex did not appear as significant features in metabolites-based hypertension classification. In Set C vs. Set D, bifurcation based on patients’ sex, higher accuracy was observed for CS vs. PHT in Set D (~73%) compared to Set C (~64%). However, the specificities for Set D were lower than Set C. Also, the corresponding sensitivities for Set D were higher than those compared to Set C. For EHT vs. PHT, PA vs. PHT, and PPGL vs. PHT, Set C had consistently higher accuracies than Set D except for a few classifiers in PPGL vs. PHT. The sensitivities for EHT vs. PHT, PA vs. PHT, and PPGL vs. PHT were higher for the female set (Set D) in comparison to the male set (Set C). The accuracies, sensitivities, and F1 scores for All vs. All were very low for both sets, however, the corresponding specificities were high.

Next, Set E was compared to Set F, where higher accuracies and AUC were observed for younger patients (Set F) only for CS vs. PHT. For other disease combinations, older patients (Set E) had higher accuracies. The specificities for CS vs. PHT and PPGL vs. PHT were higher for Set F than Set E, but opposite in the case of all other disease combinations. Overall, higher sensitivities were observed for EHT vs. PHT in Set F than Set E.

#### 3.2.2. Discriminating Features

**Figure 3:**
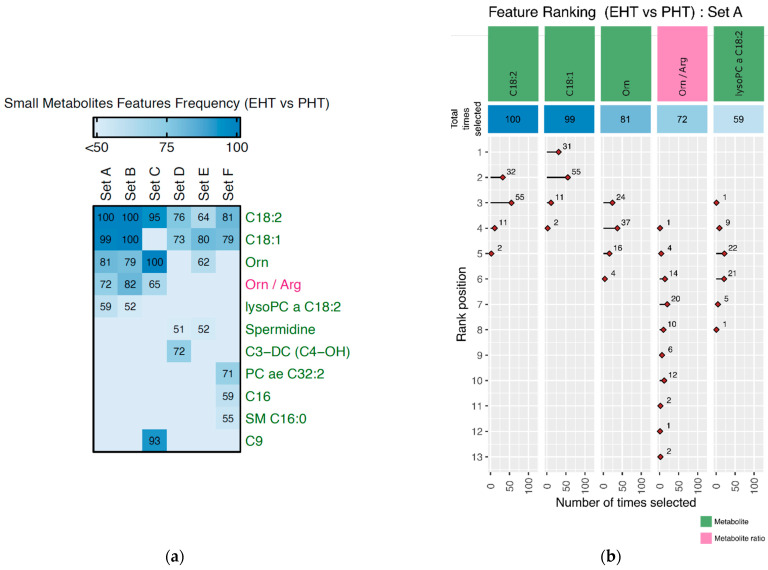
(**a**) Heatmap showing the number of times a feature (metabolites or its ratios) was selected for EHT vs. PHT disease comparison in different sets (A–F). (**b**) Feature ranking for Set A in EHT vs. PHT disease comparison.

Figure 3a shows the list of important metabolites (in green) and metabolite ratios (in pink) with the most common on top and used >50 times during MCCV for various sets within EHT vs. PHT disease classification. C18:1 and C18:2 were the two most prominent features for almost all sets except Set C. Almost similar features were selected for Set A and B. However, for Set C and D, Orn, Orn/Arg, and C9 were not selected for Set D, while C3-DC (C4-OH) was not selected for Set C. Notably, C9 was prominently selected only in Set C and not any other Set. In the case of Set F, three metabolites (C16, SM C16:0, and PC ae C32:2) were selected, which did not appear as prominent in any of the other Sets. On the other hand, Set E Spermidine was selected along with C18:1, C18:2, and Orn.

**Figure A1:**
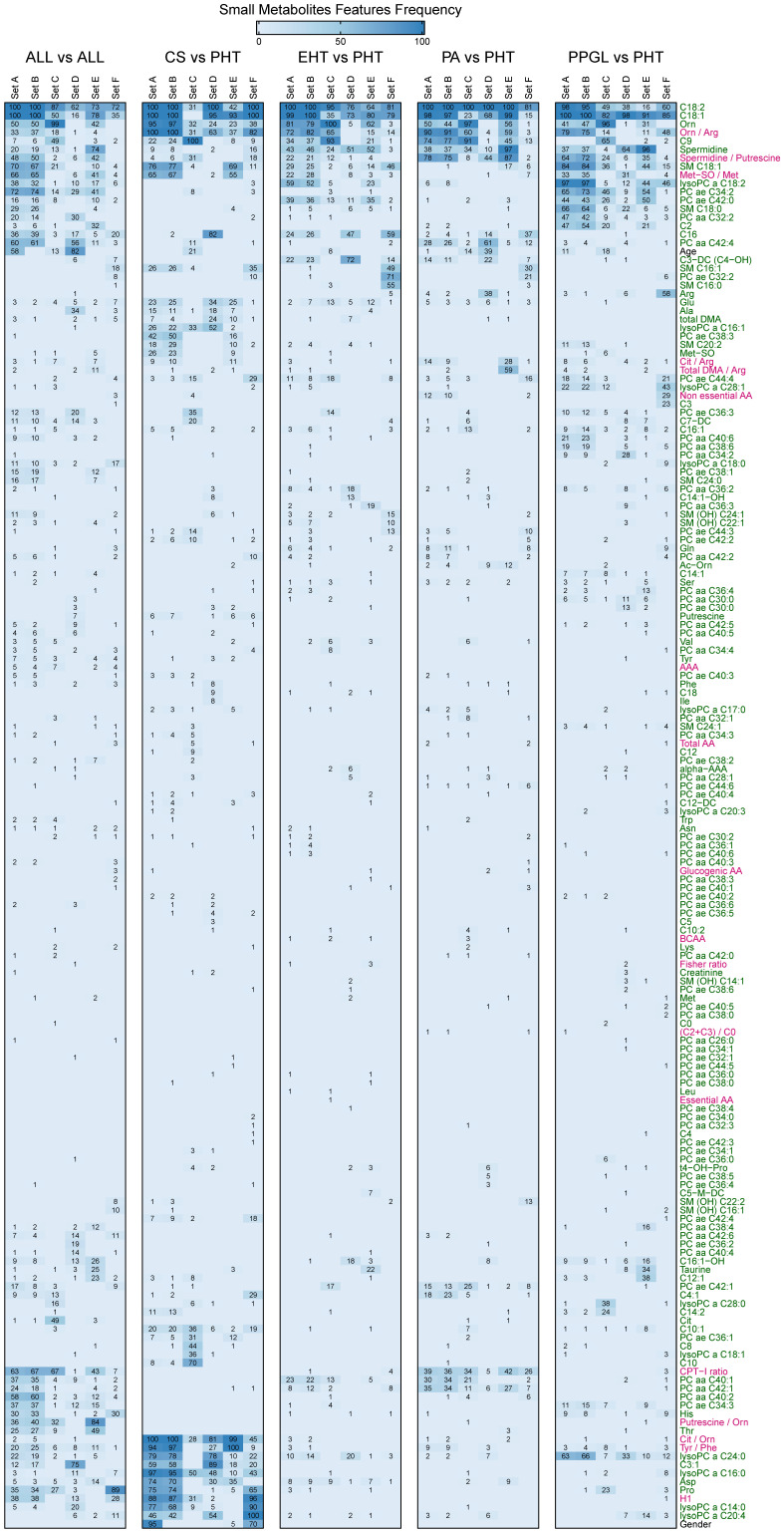
Combined heatmap showing the number of times featured for Sets A–F, showing all metabolites (in green) and metabolite ratios (in pink) selected for all 5 disease combinations.

Figure A1 in Appendix A shows a combined summary list of all features used for classifying the remaining disease combinations for all given sets (Set A–F).

**Figure A2:**
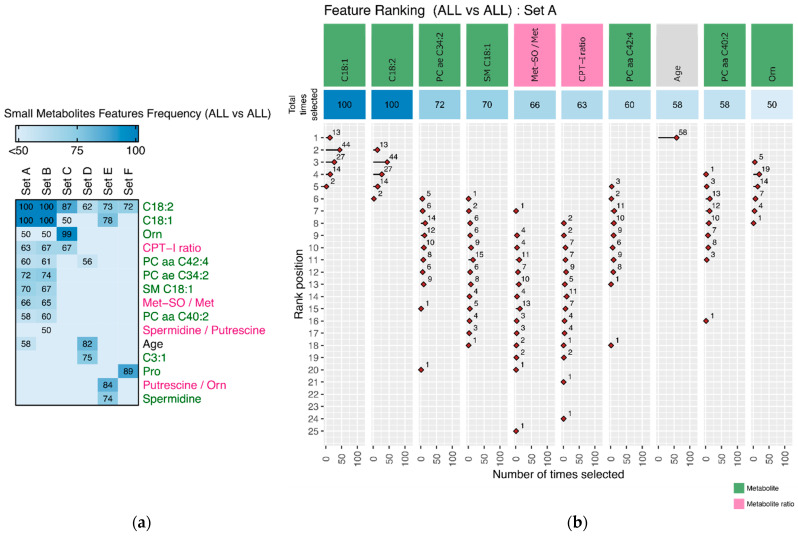
(**a**) Heatmap showing the number of times a feature (metabolites or its ratios) was selected for ALL vs. ALL disease comparison in different sets (A–F); (**b**) Feature ranking for Set A in ALL vs. ALL disease comparison.

**Figure A3:**
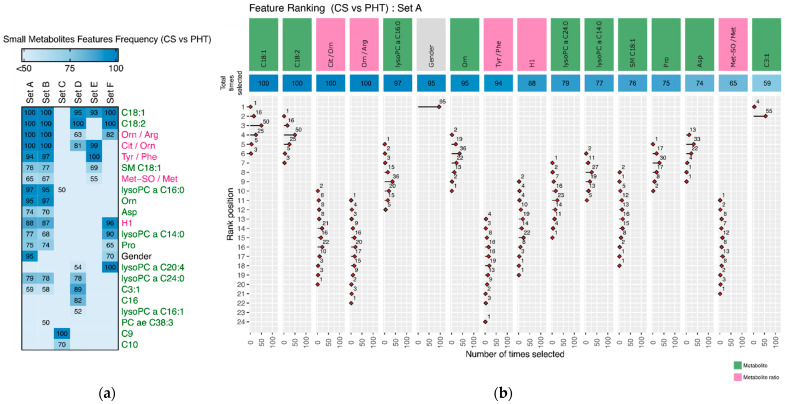
(**a**) Heatmap showing the number of times a feature (metabolites or its ratios) was selected for CS vs. PHT disease comparison in different sets (A–F); (**b**) Feature ranking for Set A in CS vs. PHT disease comparison.

**Figure A4:**
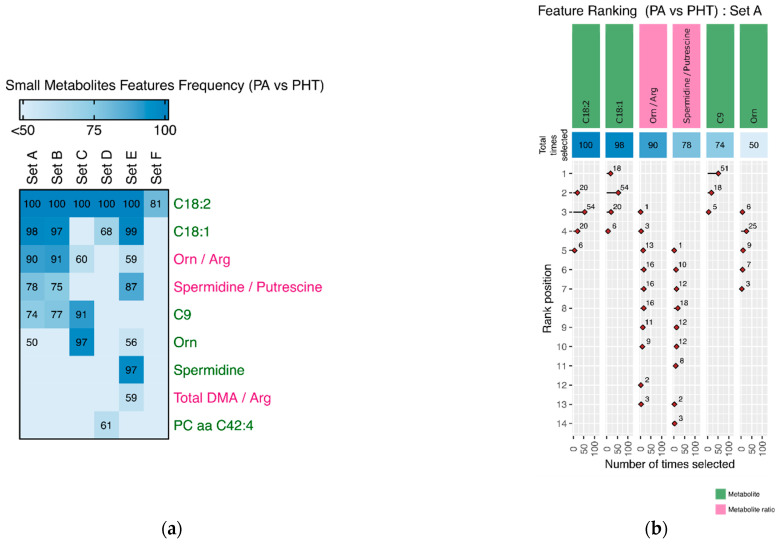
(**a**) Heatmap showing the number of times a feature (metabolites or its ratios) was selected for PA vs. PHT disease comparison in different sets (A–F); (**b**) Feature ranking for Set A in PA vs. PHT disease comparison.

**Figure A5:**
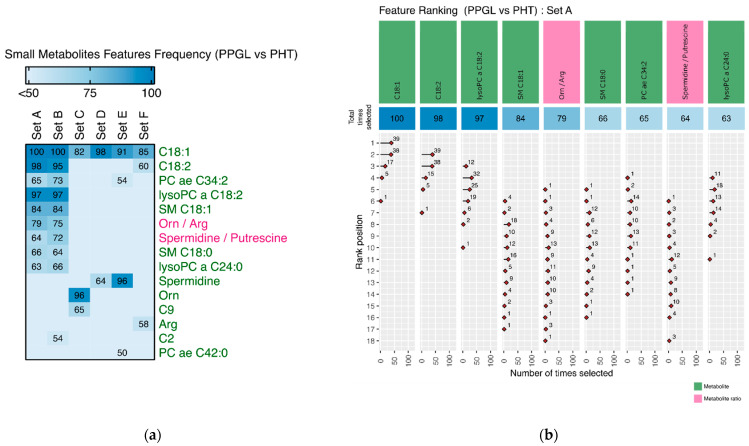
(**a**) Heatmap showing the number of times a feature (metabolites or its ratios) was selected for PPGL vs. PHT disease comparison in different sets (A–F); (**b**) Feature ranking for Set A in PPGL vs. PHT disease comparison.

Figure 3b shows rank details of selected features during 100 MCCV repeats for EHT vs. PHT disease classification based on Set A. Metabolite C18:2 was selected during all 100 MCCV repeats and ranked as second for 32 times, third for 55 times followed by 11 and 2 times in position four and four, respectively. Similarly, C18:1 was selected 99 times, however, it was ranked first 31 times and second 55 times, followed by 11 and 2 times. This indicates that although C18:2, it was selected more times than C18:1. However, still C18:1 was ranked higher 31 times in comparison to C18:2. In the case of Orn, Orn/Arg, and lysoPC, of C18:2, they are selected as 81, 72, and 59 times, respectively. Amongst the three, Orn was ranked higher consistently (rank third and fourth) and therefore should be considered more important due to its higher ranking. The ranking of all selected features and their frequency of selection during 100 MCCV thus provides a robust evaluation of the prominent discriminating features in disease classification. The corresponding results for the other four disease comparisons were shown in Appendix A (Figure A2, Figure A3, Figure A4 and Figure A5).

### 3.3. Final Model Training and Testing

In Phase 3 of the ML pipeline, the training set based on the list of selected features (from Phase 2) is used to train the best classifier (from Phase 1). Table 3 shows the classification results on the test set for the five disease combinations using the best-performing classifier. It also shows the distribution of the reduced feature set along with the balanced accuracy, sensitivity, specificity, F1 score, and AUC. CS vs. PHT provided the best classification (balanced accuracy: 83%, sensitivity: 75%, specificity: 92%) on the test set using the LMT classifier with a reduced set of 22 features (16 metabolites and 5 metabolite ratios and sex). Similarly, for EHT vs. PHT, 92% specificity was achieved although balanced accuracy, and specificity was 74% and 57%, respectively.

In terms of age and sex as features, it is evident that age and sex were only selected for ALL vs. ALL and CS vs. PHT respectively and were not used for the training of the remaining three disease combinations’ classifiers.

Finally, Table 4 shows the confusion matrix for the classification using the test set for CS vs. PHT disease combination. The values in the diagonal position show the number of correctly classified patients. For example, for CS vs. PHT, 6 CS and 11 PHT patients were correctly classified; however, in total three patients were misclassified. Table A7, Table A8, Table A9 and Table A10 show the confusion matrices for the test sets of the remaining four disease combinations.

## 4. Discussion

The application of machine learning has recently facilitated the use of high-throughput omics technologies in healthcare. In this study, we investigate the use of targeted metabolomics data for classifying and distinguishing the various subtypes of endocrine and primary hypertension using machine learning methods. From a clinical perspective, discriminating individuals with endocrine hypertension from primary hypertension is a challenging task that often involves intensive medical work-up and imaging protocols (See details in Appendix B). However, this study used a data-driven approach for identifying metabolomic patterns that can provide further insight into different hypertension subtypes without any other a priori information.

We investigated a range of disease comparisons in different sets using three feature selection methods and eight classifiers with the MCCV approach. Amongst the three feature selection methods, Boruta outperformed others in terms of classification performance as it is a wrapper-based method that detects interactions between features during selection. It evaluates the most optimal subset of features using its importance scoring mechanism [41]. On the other hand, CFS is a filter-based method that does not consider relationships between features during selection. Out of eight, four classifiers (LB, LMT, RF, and SL) provided better performance amongst all while using the same selected metabolomic features.

Our current results correspond well with our preliminary results [6] and also provide a more detailed and insightful feature ranking for each disease classification. For example, in the case of EHT vs. PHT, the common top metabolomic features were C18:2, C18:1, C9, C16, ornithine, spermidine, and ornithine/arginine, pointing to our possible association of acylcarnitine and bioamine metabolic disturbances in the pathogenesis of the morbidity and cardiovascular complications in patients with EHT, as discussed in our previous work [6]. Similarly, for other disease comparisons, distinct discriminating features emerged that can be further investigated. In particular, elevated long-chain acylcarnitines (e.g., C18:1, C18:2) have been observed in patients with heart failure and have been shown to play a role in disrupting cardiac electrophysiology and cell contractility as well as being associated with insulin resistance and diabetes mellitus. The identified amino acids and biogenic amines alterations in patients with endocrine hypertension may be related to increased inflammation and endothelial dysfunction, all of which may contribute together to the increased cardiovascular morbidity observed in EHT compared with PHT, as discussed previously [6]. Further studies are needed to clarify whether these findings are associated with a common pathogenic mechanism or are related to EHT. Instead of using a standardised ML pipeline, this work utilised a novel approach that used three phases to find a robust list of selected metabolomic features, which were used for model training and then evaluated on the test set. The selected features are not considered just based on their random repeat frequency but rather on the number of times a feature is selected along with its ranking, which provides greater insight into the most discriminating features. It was interesting to identify the variation in selected features based on the age of patients. For example, in the case of EHT vs. PHT disease combination, alongside common features (C18:1 and C18:2), a different combination of unique features was selected for patients younger than 50 years of age.

This machine learning-based study had few limitations. Firstly, class imbalance was observed in the acquired dataset. For example, fewer CS patients, since it is a rarer disease. To balance the classifier training, a downsampling approach was adopted, which led to the loss of samples from the majority class. This strong natural disbalance between different aetiologies can be improved in future by using advanced oversampling techniques such as Synthetic Minority Over-sampling TEchnique (SMOTE) [42] for ML model training. Secondly, due to the unavailability of an independent test dataset, the dataset was randomly partitioned into a training/testing dataset for MCCV (with 100 random repeats) approach for an extended validation. The reported results are based on the limited size of the cohort. Further, sensitivity for discrimination was not optimal in all subgroup analyses; it was best in discriminating EHT from PHT. Thus, while we were able to confirm the results of our previous work that our approach could potentially be used as a pre-screening test to identify patients requiring further endocrine testing by a specialist, namely the EHT group [6], it is not suitable for distinguishing the different endocrine entities from each other due to its low sensitivity (Figure 2). Finally, within our study, we did not differentiate between distinct aetiologies of the hormonal excess in the EHT cases (e.g., adrenal or pituitary cause of cortisol excess, bilateral or unilateral PA).

While clinical presentation, further diagnostic procedures, and treatment will be dependent on the final diagnosis, the overall aim of this study was to evaluate the use of metabolites and their ratios for developing a prediction tool to distinguish the endocrine hypertension forms from primary hypertension as a first screening step in the evaluation of hypertension patients. The subtype classification of the aetiology of hormonal excess in endocrine hypertension cases was considered out of scope at this stage, however, in future studies, it would be interesting to analyse the potential of metabolomics for this purpose. Another study (currently in progress) with a larger prospective dataset would further help in understanding the top discriminating features and allow refinement of the machine learning-based modelling. In future prospective studies, it will be also of interest to analyse the role of metabolomics as a prognostic factor e.g., medical treatment outcome or risk of cardiovascular events in patients with arterial hypertension. Similarly, the most recently studied TroponinT, which is a widely used diagnostic marker for cardiac ischemia, has shown a promising role as a marker for predicting cardiac surgery outcomes [43].

## 5. Conclusions

This study classified different hypertension subtypes using targeted metabolomics and their ratios. The ML pipeline comprised of five disease comparisons and nine supervised learning algorithms that used different age and sex-based sets. Amongst all the different disease combinations, CS vs. PHT and EHT vs. PHT provided the highest specificity (92%) on the test dataset using LMT and RF classifiers respectively. The evaluation showed promising results with a reduced set of features, which can be further investigated in the future on a much larger prospective dataset.

## Figures and Tables

**Table 1:** Patient data for all disease types namely Cushing’s syndrome (CS), primary aldosteronism (PA), pheochromocytoma or paraganglioma (PPGL), and primary hypertension (PHT). There was a significant difference in the distribution of patients according to sex (*p* < 0.001) and age (*p* = 0.006) between the disease groups. The difference was significant also when considering CS, PA, and PPGL in the common EHT group for sex (*p* = 0.009), but not for age (*p* = 0.088). For distribution difference analysis, the Pearson Chi-Square Test was performed using the SPSS^®^ Statistics v26.0 (IBM).

Disease	Patient Count (*n*=)	Sex		Age Distribution	
		Male (*n*=)	Female (*n*=)	Patient Age ≥ 50	Patient Age < 50
Cushing’s Syndrome (CS)	40	4	36	22	18
Primary Aldosteronism (PA)	107	58	49	42	65
Pheochromocytoma or Paraganglioma (PPGL)	76	33	43	48	28
Primary Hypertension (PHT)	59	40	19	23	36

**Table 2:** Mean balanced accuracy, sensitivity, and specificity (across the 100 MCCV repeats) for ALL vs. ALL disease combinations for all 9 classifiers using all features, CFS, and Boruta methods.

	ALL vs. ALL														
Classifier	All					CFS						Boruta			
	B. Acc (%)	Sen (%)	Spec (%)	F1	AUC	B. Acc (%)	Sen (%)	Spec (%)	F1	AUC	B. Acc (%)	Sen (%)	Spec (%)	F1	AUC
IBk	60	41	79	0.39	0.60	57	35	78	0.29	0.57	58	37	79	0.35	0.58
J48	56	35	78	0.30	0.58	57	36	78	0.31	0.60	56	34	78	0.27	0.57
LB	61	42	80	0.41	0.71	60	40	80	0.31	0.68	60	40	80	0.32	0.68
LMT	69	54	84	0.53	0.81	58	38	79	0.32	0.69	60	41	80	0.36	0.69
NB	64	48	81	0.44	0.73	59	40	79	0.26	0.68	60	41	80	0.29	0.68
RF	60	40	80	0.24	0.76	59	38	79	0.29	0.68	59	38	79	0.28	0.70
SL	69	54	84	0.54	0.82	58	38	79	0.31	0.69	60	41	80	0.35	0.70
SMO	71	56	85	0.57	0.78	51	27	76	0.2	0.63	54	31	77	0.06	0.64

**Table 3:** Classification results for disease comparisons showing balanced accuracy, sensitivity, specificity, F1 score, and AUC for the test set (Phase 3). It includes the breakdown of features and highlights whether age and sex were selected amongst them.

Disease Comparisons	Classifier	Features Used					B. Accuracy (%)	Sensitivity (%)	Specificity (%)		
		Age Included?	Sex Included?	No of Metabolites	No of Metabolite Ratios	Total				F1	AUC
PA vs. PHT	SL	✕	✕	6	3	9	73	71	75	0.8	0.7
CS vs. PHT	LMT	✕	✔	16	5	22	83	75	92	0.8	0.8
PPGL vs. PHT	LB	✕	✕	13	2	15	78	80	75	0.8	0.8
EHT vs. PHT	RF	✕	✕	10	1	11	74	57	92	0.7	0.8
ALL vs. ALL	LMT	✔	✕	10	4	15	61	42	81	0.4	0.7

**Table 4:** Confusion matrix showing the actual and predicted labels for CS vs. PHT.

		Reference	
		CS	PHT
Prediction	CS	6	1
	PHT	2	11

## Data Availability

Data generated or analyzed during this study are included in this published article. Some datasets generated during and/or analyzed during the current study are not publicly accessible but are available from the corresponding author on reasonable request.

## Appendix A. Tables and Figures

**Table A1:** List of metabolites measured with the AbsoluteIDQ^®^ p180 Kit GAC, Helmholtz Zentrum München. Note: Complete list of the 188 metabolites. With the asterisk (*) are marked the 33 metabolites excluded after selection as described in the method section. With the double-asterisk (**) are marked 8 metabolites included in the analyses for which only the variance between batches, but not within the batches, were only slightly above the predetermined cutoff prior normalization. Abbreviations: Cx:y indicates the lipid chain composition, where “x” is the number of carbons and “y” the number of double bonds. LysoPC, lysophosphatidylcholine, PC, phosphatidylcholine; a, acyl; aa, diacyl; ae, acyl-alkyl; SM, sphingomyelin; SM(OH), hydroxysphingomyelin.

Acylcarnitines (40)			
Abbreviation	Full-Name	Abbreviation	Full-Name
C0	Carnitine	C10:1	Decenoylcarnitine
C2	Acetylcarnitine	C10:2	Decadienylcarnitine
C3	Propionylcarnitine	C12	Dodecanoylcarnitine
C3:1 **	Propenoylcarnitine	C12:1	Dodecenoylcarnitine
C3-OH *	Hydroxypropionylcarnitine	C12-DC **	Dodecanedioylcarnitine
C4	Butyrylcarnitine	C14	Tetradecanoylcarnitine
C4:1	Butenoylcarnitine	C14:1	Tetradecenoylcarnitine
C4-OH (C3-DC)	Hydroxybutyrylcarnitine	C14:1-OH	Hydroxytetradecenoylcarnitine
C5	Valerylcarnitine	C14:2	Tetradecadienylcarnitine
C5:1 *	Tiglylcarnitine	C14:2-OH *	Hydroxytetradecadienylcarnitine
C5:1-DC *	Glutaconylcarnitine	C16	Hexadecanoylcarnitine
C5-DC (C6-OH) *	Glutarylcarnitine (Hydroxyhexanoylcarnitine)	C16:1	Hexadecenoylcarnitine
C5-M-DC **	Methylglutarylcarnitine	C16:1-OH	Hydroxyhexadecenoylcarnitine
C5-OH (C3-DC-M) *	Hydroxyvalerylcarnitine (Methylmalonylcarnitine)	C16:2 *	Hexadecadienylcarnitine
C6 (C4:1-DC) *	Hexanoylcarnitine (Fumarylcarnitine)	C16:2-OH *	Hydroxyhexadecadienylcarnitine
C6:1 *	Hexenoylcarnitine	C16-OH *	Hydroxyhexadecanoylcarnitine
C7-DC **	Pimelylcarnitine	C18	Octadecanoylcarnitine
C8	Octanoylcarnitine	C18:1	Octadecenoylcarnitine
C9	Nonanoylcarnitine	C18:1-OH *	Hydroxyoctadecenoylcarnitine
C10	Decanoylcarnitine	C18:2	Octadecadienylcarnitine
Amino Acids (21)			
Abbreviation	Full-Name	Abbreviation	Full-Name
Ala	Alanine	Lys	Lysine
Arg	Arginine	Met	Methionine
Asn	Asparagine	Orn	Ornithine
Asp	Aspartate	Phe	Phenylalanine
Cit	Citrulline	Pro	Proline
Gln	Glutamine	Ser	Serine
Glu	Glutamate	Thr	Threonine
Gly	Glycine	Trp	Tryptophan
His	Histidine	Tyr	Tyrosine
Ile	Isoleucine	Val	Valine
Leu	Leucine		
Monosaccharides (1)			
Abbreviation	Full-Name		
H1	Sum of Hexoses (including Glucose)		
Glycerophospholipids (90)			
Abbreviation	Full-Name	Abbreviation	Full-Name
lysoPC a C14:0	PC aa C34:1	PC aa C42:0	PC ae C38:2
lysoPC a C16:0	PC aa C34:2	PC aa C42:1	PC ae C38:3
lysoPC a C16:1	PC aa C34:3	PC aa C42:2	PC ae C38:4
lysoPC a C17:0	PC aa C34:4	PC aa C42:4	PC ae C38:5
lysoPC a C18:0	PC aa C36:0	PC aa C42:5	PC ae C38:6
lysoPC a C18:1	PC aa C36:1	PC aa C42:6	PC ae C40:1
lysoPC a C18:2	PC aa C36:2	PC ae C30:0	PC ae C40:2
lysoPC a C20:3	PC aa C36:3	PC ae C30:1*	PC ae C40:3
lysoPC a C20:4	PC aa C36:4	PC ae C30:2	PC ae C40:4
lysoPC a C24:0 **	PC aa C36:5	PC ae C32:1	PC ae C40:5
lysoPC a C26:0 *	PC aa C36:6	PC ae C32:2	PC ae C40:6
lysoPC a C26:1 *	PC aa C38:0	PC ae C34:0	PC ae C42:0
lysoPC a C28:0 **	PC aa C38:1 *	PC ae C34:1	PC ae C42:1
lysoPC a C28:1 **	PC aa C38:3	PC ae C34:2	PC ae C42:2
PC aa C24:0 *	PC aa C38:4	PC ae C34:3	PC ae C42:3
PC aa C26:0	PC aa C38:5	PC ae C36:0	PC ae C42:4
PC aa C28:1	PC aa C38:6	PC ae C36:1	PC ae C42:5
PC aa C30:0	PC aa C40:1	PC ae C36:2	PC ae C44:3
PC aa C30:2 *	PC aa C40:2	PC ae C36:3	PC ae C44:4
PC aa C32:0	PC aa C40:3	PC ae C36:4	PC ae C44:5
PC aa C32:1	PC aa C40:4	PC ae C36:5	PC ae C44:6
PC aa C32:2 **	PC aa C40:5	PC ae C38:0	
PC aa C32:3	PC aa C40:6	PC ae C38:1	
Sphingolipids (15)			
Abbreviation	Full-Name	Abbreviation	Full-Name
SM (OH) C14:1	SM C18:0	SM (OH) C22:1	SM (OH) C24:1
SM C16:0	SM C18:1	SM (OH) C22:2	SM C26:0 *
SM C16:1	SM C20:2	SM C24:0	SM C26:1 *
SM (OH) C16:1	SM C22:3 *	SM C24:1	
Biogenic Amines (21)			
Abbreviation	Full-Name	Abbreviation	Full-Name
Ac-Orn	Acetylornithine	PEA *	Phenylethylamine
ADMA *	Asymmetric dimethylarginine	cis-OH-Pro *	cis-4-Hydroxyproline
alpha-AAA	alpha-Aminoadipic acid	trans-OH-Pro	trans-4-Hydroxyproline
Carnosine *	Carnosine	Putrescine	Putrescine
Creatinine	Creatinine	SDMA *	Symmetric dimethylarginine
DOPA *	DOPA	Serotonin *	Serotonin
Dopamine *	Dopamine	Spermidine	Spermidine
Histamine *	Histamine	Spermine *	Spermine
Kynurenine *	Kynurenine	Taurine	Taurine
Met-SO	Methionine sulfoxide	total DMA	Total dimethylarginine
Nitro-Tyr *	Nitrotyrosine		

**Table A2:** Details of randomly partitioned training and testing datasets.

Data	Disease	Sex		Age Distribution		Total Count
		Male	Female	Patient Age ≥ 50	Patient Age < 50	
Training (80%)	CS	3	29	17	15	32
	PA	45	41	33	53	86
	PPGL	27	34	39	22	61
	PHT	29	18	22	25	47
Testing (20%)	CS	1	7	5	3	8
	PA	13	8	9	12	21
	PPGL	6	9	9	6	15
	PHT	11	1	1	11	12

**Table A3:** Mean balanced accuracy, sensitivity, and specificity for EHT vs. PHT disease comparison using various classifiers with all features, CFS, and Boruta feature selection methods.

	EHT vs. PHT														
Classifier	All					CFS					Boruta				
	B. Acc (%)	Sen (%)	Spec (%)	F1	AUC	B. Acc (%)	Sen (%)	Spec (%)	F1	AUC	B. Acc (%)	Sen (%)	Spec (%)	F1	AUC
IBk	61	83	39	0.84	0.61	62	80	44	0.82	0.62	58	81	36	0.82	0.58
J48	58	83	34	0.83	0.56	56	85	27	0.83	0.58	56	86	25	0.84	0.63
LB	61	89	33	0.87	0.74	59	89	30	0.86	0.74	59	88	29	0.86	0.75
LMT	62	91	33	0.87	0.76	56	93	18	0.87	0.70	55	92	19	0.86	0.69
NB	70	62	78	0.74	0.76	72	61	83	0.74	0.78	68	56	81	0.70	0.76
RF	53	99	7	0.89	0.77	58	94	22	0.88	0.75	57	90	24	0.86	0.74
SL	61	91	31	0.88	0.76	55	94	16	0.87	0.70	54	93	16	0.87	0.69
SMO	62	91	33	0.87	0.62	50	100	0	0.89	0.50	50	100	0	0.89	0.50

**Table A4:** Mean balanced accuracy, sensitivity, and specificity for CS vs. PHT disease comparison using various classifiers with all features, CFS, and Boruta feature selection methods.

	CS vs. PHT														
Classifier	All					CFS					Boruta				
	B. Acc (%)	Sen (%)	Spec (%)	F1	AUC	B. Acc (%)	Sen (%)	Spec (%)	F1	AUC	B. Acc (%)	Sen (%)	Spec (%)	F1	AUC
IBk	82	73	91	0.77	0.82	83	74	91	0.78	82	0.87	80	94	0.84	0.87
J48	76	73	78	0.71	0.75	74	70	78	0.68	74	0.74	71	78	0.69	0.74
LB	75	66	84	0.69	0.85	76	66	86	0.70	85	0.76	67	85	0.70	0.85
LMT	83	75	91	0.79	0.92	82	74	90	0.77	91	0.82	74	90	0.78	0.92
NB	81	74	88	0.76	0.87	81	67	95	0.75	91	0.83	70	96	0.78	0.94
RF	77	60	95	0.70	0.92	78	65	91	0.71	89	0.79	65	92	0.73	0.90
SL	83	75	91	0.79	0.92	82	74	90	0.77	91	0.82	74	90	0.78	0.91
SMO	87	82	93	0.84	0.87	81	69	93	0.76	81	0.83	70	95	0.78	0.83

**Table A5:** Mean balanced accuracy, sensitivity, and specificity for PA vs. PHT disease comparison using various classifiers with all features, CFS, and Boruta feature selection methods.

	PA vs. PHT														
Classifier	All					CFS					Boruta				
	B. Acc (%)	Sen (%)	Spec (%)	F1	AUC	B. Acc (%)	Sen (%)	Spec (%)	F1	AUC	B. Acc (%)	Sen (%)	Spec (%)	F1	AUC
IBk	63	72	55	0.73	0.63	60	66	54	0.69	0.60	62	69	55	0.71	0.62
J48	63	72	54	0.73	0.64	64	70	59	0.73	0.66	65	72	59	0.74	0.67
LB	65	76	53	0.76	0.74	65	78	52	0.76	0.75	65	76	54	0.76	0.75
LMT	67	77	56	0.77	0.78	66	75	57	0.75	0.77	66	76	57	0.76	0.77
NB	69	57	81	0.68	0.75	73	59	88	0.70	0.79	72	56	87	0.68	0.78
RF	62	88	37	0.79	0.78	65	78	52	0.77	0.76	64	77	51	0.76	0.75
SL	67	77	56	0.77	0.78	66	75	57	0.76	0.78	67	76	58	0.76	0.78
SMO	70	77	62	0.78	0.70	59	84	35	0.76	0.59	58	88	29	0.78	0.58

**Table A6:** Mean balanced accuracy, sensitivity, and specificity for PPGL vs. PHT disease comparison using various classifiers with all features, CFS, and Boruta feature selection methods.

	PPGL vs. PHT														
Classifier	All					CFS					Boruta				
	B. Acc (%)	Sen (%)	Spec (%)	F1	AUC	B. Acc (%)	Sen (%)	Spec (%)	F1	AUC	B. Acc (%)	Sen (%)	Spec (%)	F1	AUC
IBk	62	54	71	0.61	0.62	66	63	70	0.67	0.66	65	64	66	0.67	0.65
J48	66	71	62	0.71	0.66	66	72	60	0.71	0.67	68	73	63	0.72	0.69
LB	70	74	67	0.74	0.78	71	75	67	0.75	0.80	74	79	69	0.78	0.82
LMT	71	73	69	0.75	0.79	69	73	66	0.73	0.76	69	74	65	0.73	0.76
NB	73	67	79	0.73	0.81	73	64	82	0.72	0.81	70	59	80	0.68	0.79
RF	73	84	62	0.79	0.83	73	79	67	0.77	0.81	74	79	68	0.78	0.82
SL	72	74	70	0.75	0.79	70	73	67	0.73	0.76	70	74	65	0.73	0.77
SMO	74	79	68	0.78	0.74	71	74	68	0.75	0.71	70	73	66	0.74	0.70

**Table A7:** Confusion matrix showing the actual and predicted labels for PA vs. PHT.

		Reference	
		PA	PHT
Prediction	PA	15	3
	PHT	6	9

**Table A8:** Confusion matrix showing the actual and predicted labels for PPGL vs. PHT.

		Reference	
		PPGL	PHT
Prediction	PPGL	12	3
	PHT	3	9

**Table A9:** Confusion matrix showing the actual and predicted labels for EHT vs. PHT.

		Reference	
		EHT	PHT
Prediction	EHT	25	1
	PHT	19	11

**Table A10:** Confusion matrix showing the actual and predicted labels for ALL vs. ALL.

		Reference			
		CS	PA	PHT	PPGL
Prediction	CS	2	2	0	5
	PA	0	6	2	0
	PHT	2	10	8	3
	PPGL	4	3	2	7

## Appendix B. Patient Recruitment and Diagnostic Work-Up

Patient data and suitable plasma specimen following overnight fasting were available from patients from 11 centres of the ENSAT-HT consortium (http://www.ensat-ht.eu accessed on 1 June 2022). Included were patients with the diagnosis of arterial hypertension either by use of antihypertensive medication or if untreated confirmed by daytime ambulatory blood pressure monitoring, or home blood pressure monitoring, with blood pressure higher or equal to 135 mmHg for systolic blood pressure and/or higher or equal to 85 mmHg for diastolic blood pressure. Patients were classified as primary or essential hypertension (PHT) after exclusion of primary hyperaldosteronism (PA), cathecholamin-excess due to pheochromocytoma/paraganglioma (PPGL) and Cushing syndrome (CS) (adrenal and pituitary), and other forms of secondary hypertension (renal disease, pharmacological cause and obstructive sleep apnea syndrome). CS was diagnosed in the presence of two abnormal test results of any of the following tests: urine free cortisol (UFC; at least two measurements), late-night salivary cortisol (two measurements), 1 mg overnight dexamethasone suppression test (DST), and longer low-dose DST (2 mg/d for 48 h). The diagnosis (PA, PPGL) was made according to the current guidelines for screening and management of the specific diseases [44,45]. Only patients with PHT, CS, PA, and PPGL were included in the study. Excluded were also patients with low-renin hypertension, unclear diagnosis, pregnancy, and severe comorbidities (e.g., heart failure, chronic kidney disease, active malignancy). All patients provided written consent to participate in the study according to the protocol approved by the Ethics Committee of each participating centre.

## Appendix C. Metabolite Quantification by Absolute*IDQ*^TM^ p180 Kit

For the LC-part, compound identification and quantification were based on scheduled multiple reaction monitoring measurements (sMRM). The method of Absolute*IDQ*^TM^ p180 Kit has been proven to be in conformance with the EMEA-Guideline [46], which implies proof of reproducibility within a given error range. Sample preparation and LC-MS/MS measurements were performed as described in the manufacturer in manual UM-P180. Analytical specifications for LOD and evaluated quantification ranges, further LOD for semiquantitative measurements, identities of quantitative and semiquantitative metabolites, specificity, potential interferences, linearity, precision and accuracy, reproducibility, and stability were described in Biocrates manual AS-P180. The LODs were set to three times the values of the zero samples (PBS). The assay procedures of the Absolute*IDQ*^TM^ p180 Kit as well as the metabolite nomenclature have been described in detail previously [20,21]. Sample handling was performed by a Hamilton Microlab STAR^TM^ robot (Hamilton Bonaduz AG, Bonaduz, Switzerland) and a Ultravap nitrogen evaporator (Porvair Sciences, Leatherhead, UK), beside standard laboratory equipment. Mass spectrometric analyses were done on an API 4000 triple quadrupole system (Sciex Deutschland GmbH, Darmstadt, Germany) equipped with a 1200 Series HPLC (Agilent Technologies Deutschland GmbH, Böblingen, Germany) and a HTC PAL auto sampler (CTC Analytics, Zwingen, Switzerland) controlled by the software Analyst 1.6.2. Data evaluation for quantification of metabolite concentrations and quality assessment was performed with the software MultiQuant 3.0.1 (Sciex) and the Met*IDQ*^TM^ software package, which is an integral part of the Absolute*IDQ*^TM^ Kit. Metabolite concentrations were calculated using internal standards and reported in µM.
